# Screening and identification of lncRNAs in preadipocyte differentiation in sheep

**DOI:** 10.1038/s41598-024-56091-5

**Published:** 2024-03-04

**Authors:** Zhiyun Hao, Xiayang Jin, Jon G. H. Hickford, Huitong Zhou, Longbin Wang, Jiqing Wang, Yuzhu Luo, Jiang Hu, Xiu Liu, Shaobin Li, Mingna Li, Bingang Shi, Chunyan Ren

**Affiliations:** 1 Gansu Key Laboratory of Herbivorous Animal Biotechnology, College of Animal Science and Technology, Gansu Agricultural University, Lanzhou, China; 2 Academic Animal & Veterinary Science, Qinghai University, Xining, China; 3 Gene-Marker Laboratory, Faculty of Agriculture and Life Science, Lincoln University, Lincoln, 7647 New Zealand

**Keywords:** lncRNAs, Preadipocytes, RNA-Seq, Sheep, Gene expression, Gene regulation

## Abstract

Studies of preadipocyte differentiation and fat deposition in sheep have mainly focused on functional genes, and with no emphasis placed on the role that long non-coding RNAs (lncRNAs) may have on the activity of those genes. Here, the expression profile of lncRNAs in ovine preadipocyte differentiation was investigated and the differentially expressed lncRNAs were screened on day 0 (D0), day 2(D2) and day 8(D8) of ovine preadipocyte differentiation, with their target genes being predicted. The competing endogenous RNA (ceRNA) regulatory network was constructed by GO and KEGG enrichment analysis for functional annotation, and some differentially expressed lncRNAs were randomly selected to verify the RNA-Seq results by RT-qPCR. In the study, a total of 2517 novel lncRNAs and 3943 known lncRNAs were identified from ovine preadipocytes at the three stages of differentiation, with the highest proportion being intergenic lncRNAs. A total of 3455 lncRNAs were expressed at all three stages of preadipocyte differentiation, while 214, 226 and 228 lncRNAs were uniquely expressed at day 0, day 2 and day 8, respectively. By comparing the expression of the lncRNAs between the three stages of differentiation stages, a total of 405, 272 and 359 differentially expressed lncRNAs were found in D0-vs-D2, D0-vs-D8, and D2-vs-D8, respectively. Functional analysis revealed that the differentially expressed lncRNAs were enriched in signaling pathways related to ovine preadipocyte differentiation, such as mitogen-activated protein kinase (MAPK) pathway, the phosphoinositide 3-kinase protein kinase B (PI3K-Akt) pathway, and the transforming growth factor beta (TGF-β) pathway. In summary, lncRNAs from preadipocytes at different stages of differentiation in sheep were identified and screened using RNA-Seq technology, and the regulatory mechanisms of lncRNAs in preadipocyte differentiation and lipid deposition were explored. This study provides a theoretical reference for revealing the roles of lncRNAs in ovine preadipocyte differentiation and also offers a theoretical basis for further understanding the regulatory mechanisms of ovine preadipocyte differentiation.

## Introduction

Lamb is one of the healthiest meat options since it has high contents of iron, zinc, selenium, vitamins B1, B2, and B6^1,2^. However, fat deposition directly affects the flavor and quality of meat, and it can reduce sheep productivity. For example, intramuscular fat (IMF) is a key contributor to meat flavor and palatability^3–5^. In this context, understanding the growth and differentiation of adipose tissue in sheep may aid in controlling the amount and distribution of fat deposits, with this potentially boosting consumer demand for sheep meat, thus allowing for improved market growth and development.

Lipogenesis is a complicated process that has multiple stages and complex regulatory control^6^. The differentiation of adipocytes from mesenchymal precursors (termed adipogenesis), is an important process for developing and maintaining functional adipose tissues^7^. The factors that affect adipocyte differentiation also affect fat deposition, and include genetic factors, dietary factors, and physiological conditions^6^. However, these factors likely all act through regulation of the expression of specific genes, and the modulation of various signaling pathways.

In recent years, research into lipogenesis and its regulatory mechanisms has become a focus for breeding and animal production. Researchers have identified several genes, associated proteins and transcription factors that affect preadipocyte differentiation, including the genes for the CCAAT enhancer binding proteins (C/EBPs)^8,9^, the peroxisome proliferator-activated receptor (PPAR)^10^, the early B-cell factor (EBF)^11^, the fatty acid binding proteins (FABPs)^12^, and the β-linked protein β-catenin^13^.

More recently, analysis of the genome and transcriptome has revealed a group of RNA transcripts that have been called long non-coding RNAs (lncRNAs). They appear to play an important role as epigenetic regulators^14^, and some of them have been shown to have regulatory functions in adipogenesis or lipid metabolism^15^. For example, a human study by Sun et al.^16^ analyzed the transcriptomes of primary adipocytes and identified 175 lncRNAs that were up- or down-regulated (greater than two-fold) during differentiation of both brown and white adipocytes. Some of the lncRNAs were induced during adipogenesis and bound at their promoters by key transcription factors such as PPARG and the and CCAAT/enhancer-binding protein α (C/EBPα)^16^.

Xu et al.^17^ revealed that overexpression of lncRNA steroid receptor RNA activator (SRA) in mesenchymal preadipocytes promoted their differentiation to adipocytes, and that contrastingly, knockdown of this lncRNA inhibited 3T3-L1 preadipocyte differentiation. In addition, Xiao et al.^18^ found that a lncRNA ADINR (adipose differentiation-inducing non-coding RNA), played an important role in regulating the differentiation of human mesenchymal stem cells into adipocytes by regulating *C/EBPα*. The lncRNA HOTAIR was also revealed to be able to regulate key processes of adipocyte differentiation^19^.

LncRNAs also play a central regulatory role through a competing endogenous RNA (ceRNA) regulatory mechanism. For example, a novel lncRNA-miR-140-NEAT1 ceRNA was found to be essential for adipogenesis in humans by Gernapudi et al.^20^. Specifically, miR-140 enhanced lipogenic differentiation of adipocyte-derived stem cells from mice by interacting with lncRNA-NEAT1. The knockdown of miR-140 decreased their lipogenic differentiation capacity, with these results further supporting the contention that lncRNAs play an important role in regulating fat formation. However, while some lncRNA in human adipose tissue have been revealed and their regulatory mechanisms studied, little is known about their role in the differentiation of preadipocytes in sheep.

Using RNA-Seq technology and RT-qPCR methods, lncRNAs in ovine preadipocytes were identified. Functional annotation of the target genes of the lncRNAs was analyzed using GO and KEGG enrichment analysis, and a ceRNA regulatory network was also constructed. This finding will provide data for further investigation into the regulatory function of lncRNAs in the differentiation of ovine preadipocytes.

## Results

### LncRNA expression during differentiation of ovine preadipocytes

**Figure 1:**
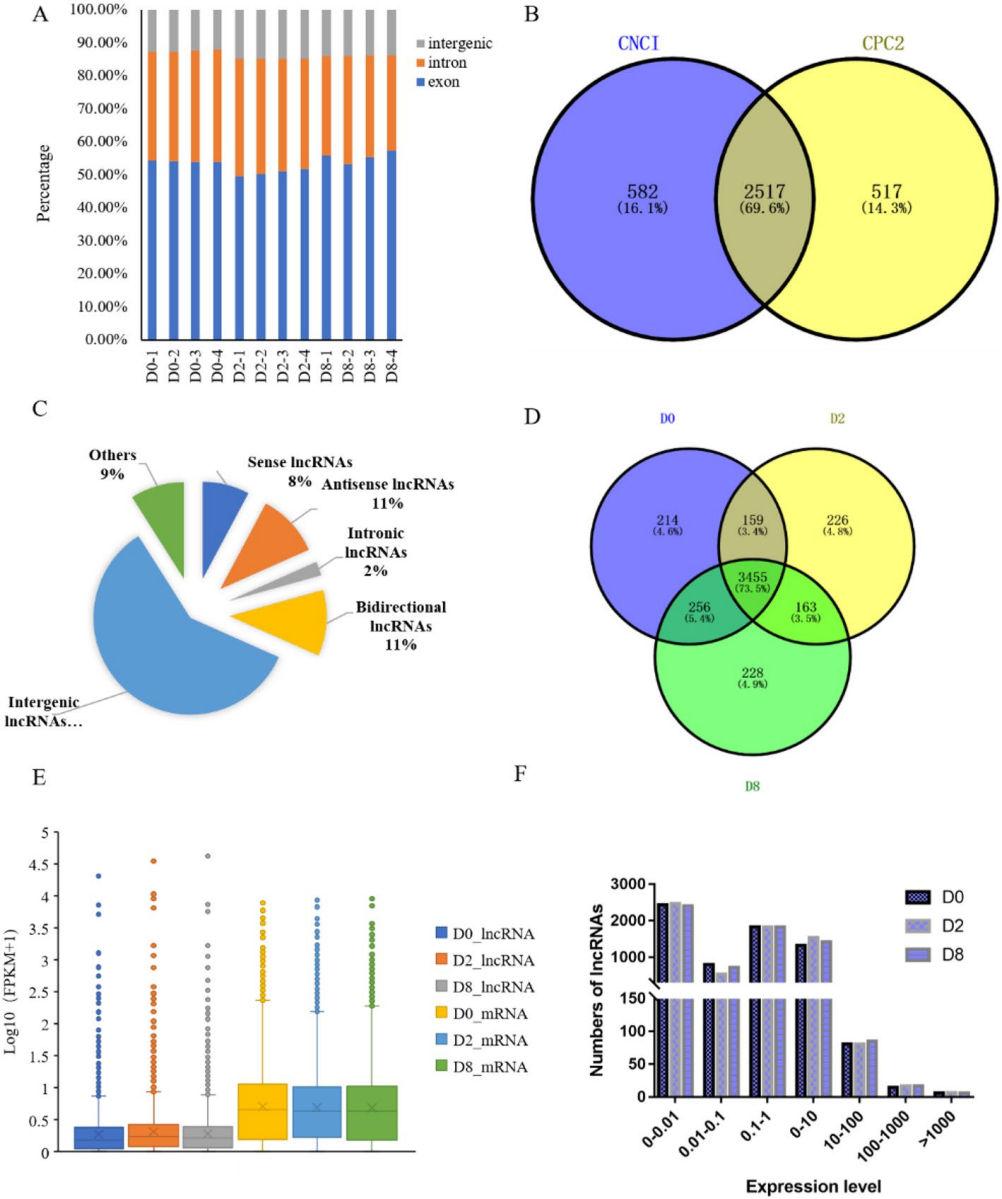
An overview of the lncRNAs that were detected during the differentiation of the ovine preadipocytes, and their expression characteristics. (**A**) The distribution of the lncRNAs in intergenic, intronic and exonic regions based on their location in the ovine reference genome v4.0. (**B**) Venn diagram summarizing the number of lncRNAs identified using the CNCI v2 and CPC v0.9-r2 software. (**C**) Summary of the type of lncRNAs identified in this study. (**D**) Venn diagram summarizing the number of lncRNAs expressed at D0, D2 and D8 in the ovine preadipocytes. (**E, F**) Comparison of expression levels of lncRNAs and mRNAs identified in the D0, D2 and D8 preadipocytes.

The raw reads from the study have been deposited into GenBank with accession numbers SRR19917845-SRR19917856. Among them, the high-quality reads obtained in all 12 samples were all above 13.5 Gb (1 Gb = 1.0 × 10^9^ Bp), and the percentage of bases with a quality score of 30 (Q30) was above 93.32% (Table 1). To further ensure the reliability of the sequencing data, a reference genome-based alignment analysis was performed using HISAT2 software (University of Texas Southwestern & Johns Hopkins University), and the clean reads of each sample were mapped to the ovine reference genome v4.0. The unique alignment ratio to the reference genome ranged from 86.64 to 89.76%, and the multiple alignment proportion to the reference genome ranged from 4.69 to 5.40%. The proportion of all clean reads that could be localized to the genome ranged from 91.65 to 94.45%. The clean reads from the 12 samples were compared with the regions of the genome. The results showed that most clean reads were found in exonic regions, followed by intronic regions. However, they were at their lowest in the intergenic regions (Fig. 1A).

**Table 1:** Summary of the RNA-Seq data after mapping to the reference genome.

Sample	Raw reads (Gb)	Q30 (%)	Clean reads (%)	Total mapped ratio (%)	Unique Mapped ratio (%)
D0	15.7	94.99	99.82	94.27	89.36
D2	14.4	95.28	99.82	92.95	87.72
D8	14.5	93.61	99.54	93.00	88.10

A total of 8819 transcripts were produced. Upon identification, there was a total of 2517 novel lncRNAs and 3943 known lncRNAs (Fig. 1B). The combined total of 6460 lncRNAs were classified into five categories based on their position in the ovine genome relative to protein-coding genes, and they included intergenic lncRNAs (59.33%), bidirectional lncRNAs (10.99%), intronic lncRNAs (2.32%), antisense lncRNAs (10.54%) and nonsense lncRNAs (7.80%) (Fig. 1C). The intergenic lncRNAs was the most common, with the number of known lncRNAs and novel lncRNAs being 2445 and 1388, respectively (Fig. 1C). Among all the lncRNAs, there were 3455 (53.48%) lncRNAs that were expressed in the preadipocytes at all three stages of differentiation (Fig. 1D), while 214, 226 and 228 lncRNAs were specifically expressed in preadipocytes at D0, D2 and D8, respectively. Among the lncRNAs that were expressed at all three stages, the novel lncRNA MSTRG.65945.1 was expressed at the highest level. It was revealed that the majority of lncRNAs detected were present at a low to medium abundance when compared to mRNA levels (Fig. 1E), with them predominantly in the fragments per kilobase of transcript per million mapped reads (FPKM) range of 0–10 (Fig. 1E,F).

**Figure 2:**
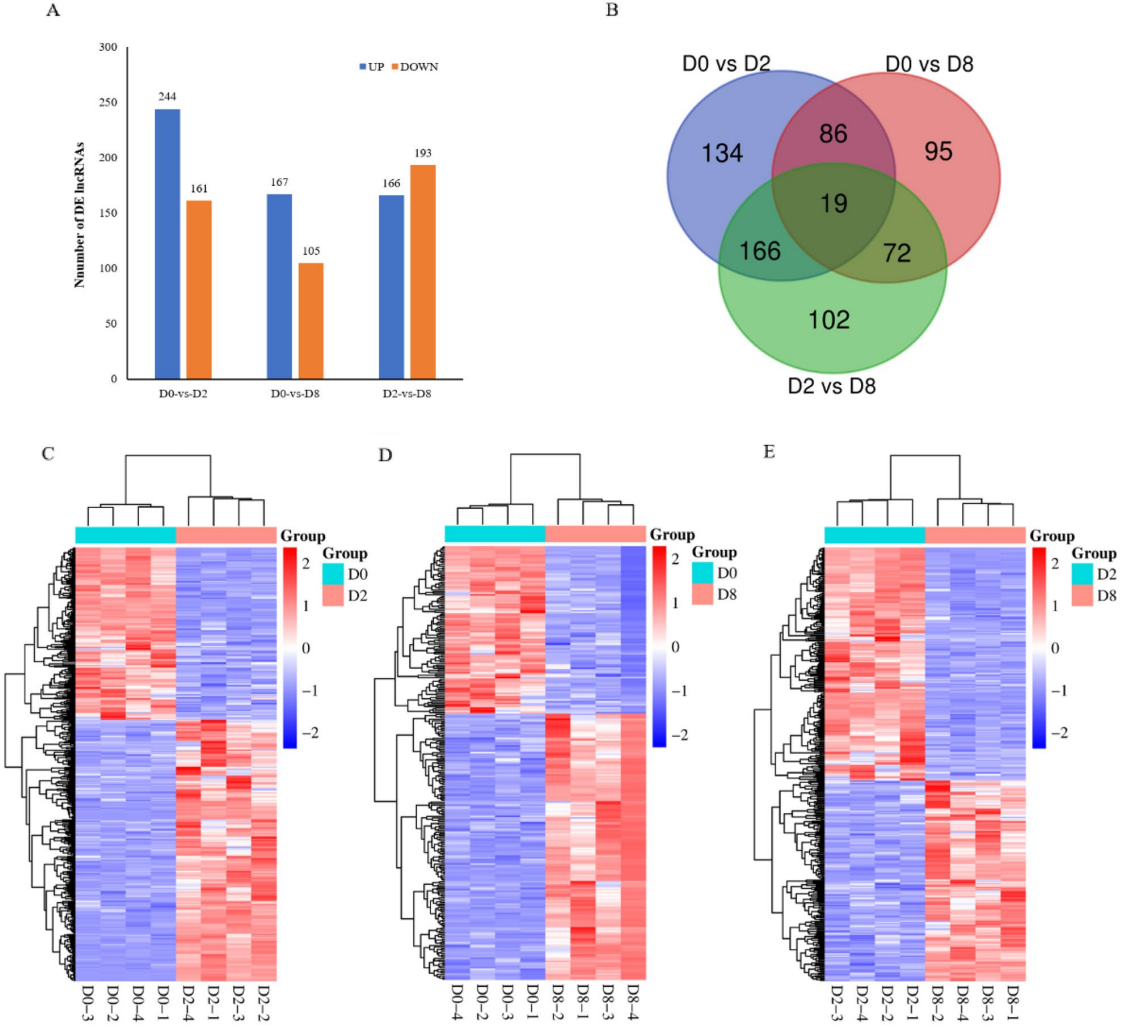
Summary of the differential expression analysis of the annotated lncRNAs. (**A**) The number of differentially expression lncRNAs. (**B**) The shared and unique lncRNAs when comparing three groups of differentially expressed lncRNAs. (**C-E**) Heat maps of differentially expressed lncRNAs were analyzed using the z-score method according to the FPKM values^21^. Blue denotes the genes with lower levels of expression, while red denotes the genes with higher levels of expression.

Based on the results of the differential analyses, 405 (244 up-regulated, 161 down-regulated), 272 (167 up-regulated, 105 down-regulated) and 359 (166 up-regulated, 193 down-regulated) differentially expressed lncRNAs were identified in D0-vs-D2, D0-vs-D8 and D2-vs-D8 comparisons, respectively (Fig. 2A). Among them, 19 lncRNAs were differentially expression in all three stages (Fig. 2B). The hierarchical clustering results of differentially expressed lncRNAs revealed a significant difference in the gene expression profiles among the three groups (Fig. 2C–E).

### GO and KEGG enrichment analyses of the target genes of the differentially expressed lncRNAs

**Figure 3:**
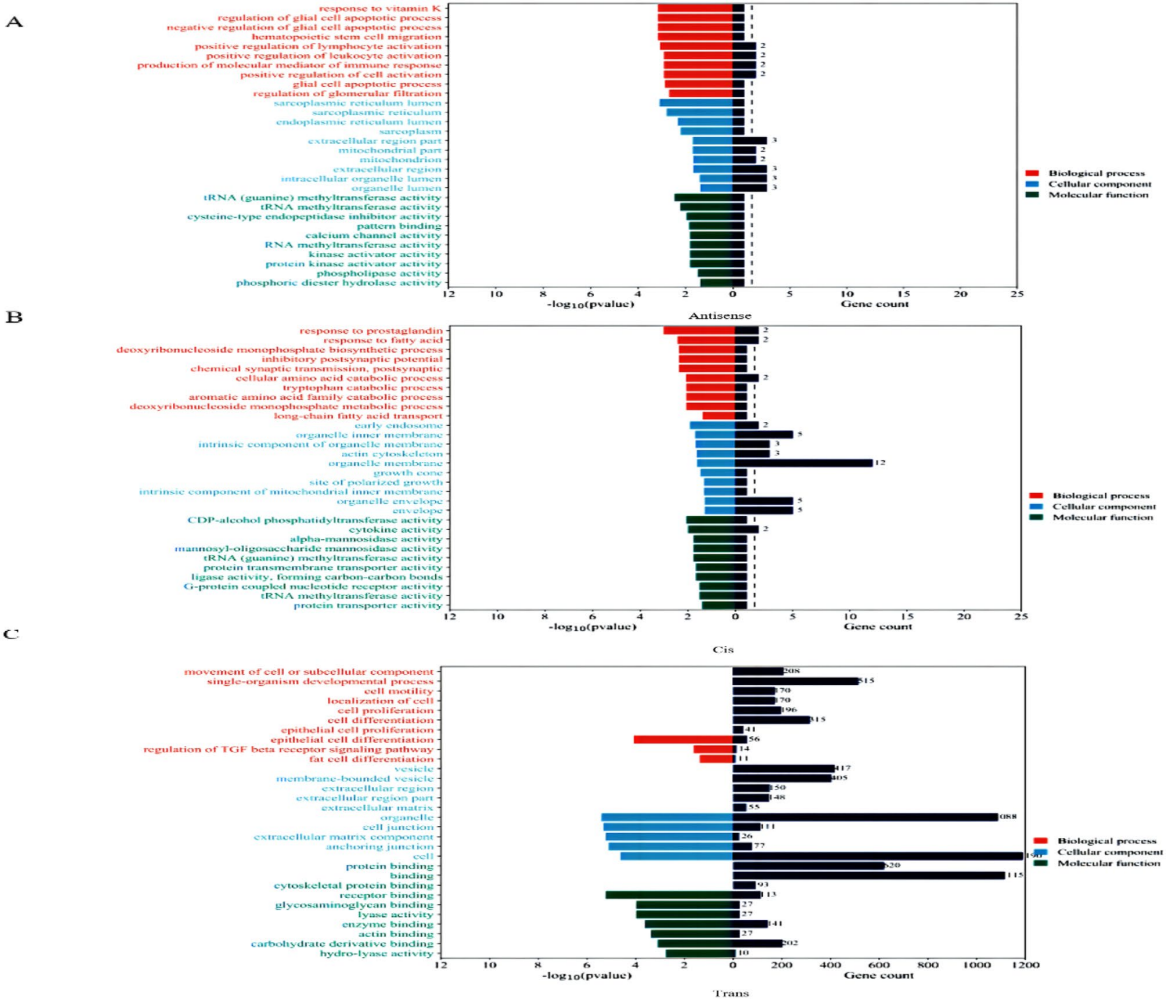
Functional categorization of the target genes of differentially expressed lncRNAs during the selected stages of differentiation of the ovine preadipocytes. (**A**) Antisense target genes. (**B**) *Cis* target genes. (**C**) *Trans* target genes.

To investigate the functions of the lncRNAs associated with ovine preadipocyte differentiation, analyses were undertaken to predict the target genes of the differentially expressed lncRNAs using antisense, *cis*-action and *trans*-action analysis. In the antisense analysis, 703 lncRNA-mRNA pairs were identified, including 14 significantly different antisense-mRNA pairs. The GO functional enrichment analysis results revealed that the target genes were mainly enriched in 544 biological processes (BP), 52 cellular components (CC), and 69 molecular functions (MF) (Fig. 3A). Most target genes were significantly enriched in the regulation of immune processes and enzyme activities, including immune responses (GO:0002440), positive regulation of lymphocyte activation (GO:0051251), phospholipase activity (GO:0004620), tRNA methyltransferase activity (GO:0008175), phosphodiester hydrolase activity (GO:0008081), cellular glucose homeostasis (GO:0001678), and response to fatty acids (GO. 0070542) (Supplementary Table 1).

In the context of a *cis* mode of action, we identified 3517 *cis*-regulatory (*cis*-mRNA) relationship pairs, of which 39 were significantly different *cis*-mRNA relationship pairs (*P* < 0.05). The GO functional enrichment analysis of the target genes revealed that they were mainly enriched in 902 GO-BP terms. Most of the target genes were enriched in GO terms related to transporter protein activity and catabolic processes, including protein transmembrane transporter protein activity (GO:0008320), protein transporter protein activity (GO:0008565), aromatic amino acid family catabolic processes (GO:0009074), long-chain fatty acid transport (GO:0015909), and cellular amino acid catabolic processes (GO:0009063) (Fig. 3B, Supplementary Table 1).

A total of 31,502 trans-mRNA pairs were identified (*P* < 0.05). The GO functional enrichment analysis results revealed that the target genes were enriched in 3772 GO-BP, 333 GO-CC and 665 GO-MF terms. The majority of target genes were mainly enriched in the regulation of cell proliferation and differentiation and protein binding, including cell proliferation (GO:0008283), regulation of TGF-β receptor signaling pathway (GO:0017015), cell differentiation (GO:0030154), epithelial cell proliferation (GO:0050673), actin binding (GO:0003779), epithelial cell differentiation (GO:0030855) and adipocyte differentiation (GO:0045444), among others (Fig. 3C, Supplementary Table 1).

**Figure 4:**
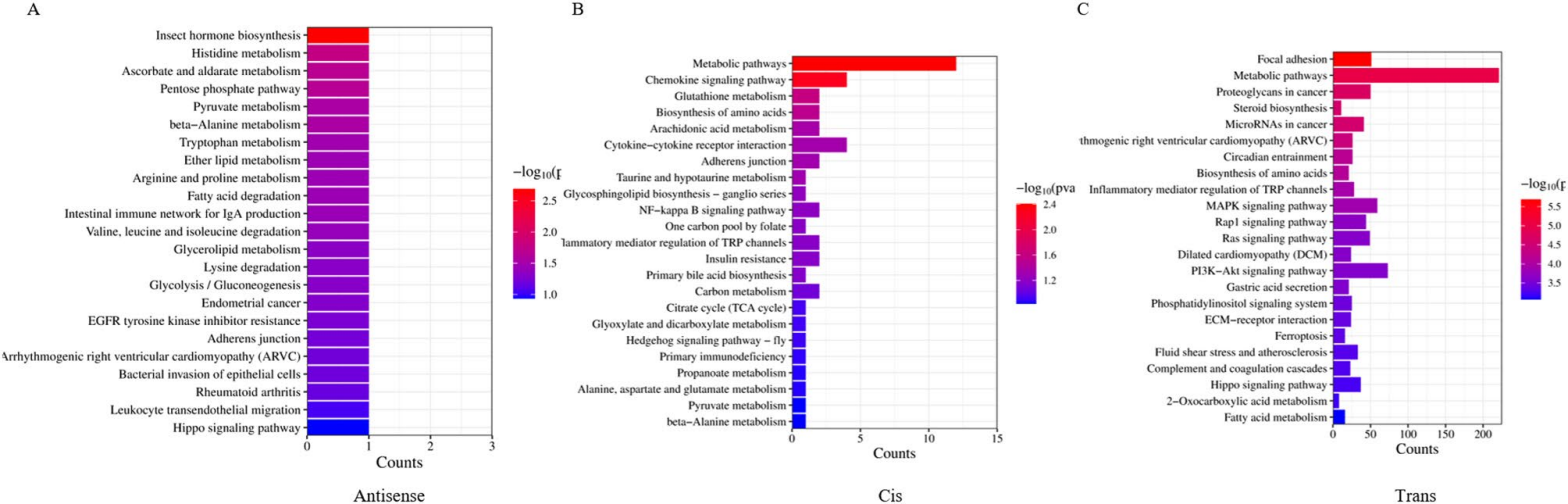
KEGG enrichment analysis of the target genes of differentially expressed lncRNAs during the differentiation stage of ovine preadipocytes. (**A**) Antisense target genes (**B**) *Cis* target genes. (**C**) *Trans* target genes.

To further analyze the possible pathways involved in ovine preadipocyte differentiation for the target genes, a KEGG enrichment analysis was undertaken. This revealed that most of antisense target genes were significantly enriched in the fatty acid degradation pathway, the glycerolipid metabolism, ether lipid metabolism, amino acid metabolism pathways, and glycolysis/gluconeogenesis signaling pathways (*P* < 0.05, Fig. 4A). Most *cis* target genes were significantly enriched in pathways including metabolic pathways, chemokine signaling pathways, amino acid biosynthesis pathways, cytokine-cytokine receptor interactions, and taurine and hypotaurine metabolism pathways (*P* < 0.05, Fig. 4B). In contrast, most *trans* target genes were significantly enriched in pathways, including the MAPK signaling pathway, the PI3K-Akt signaling pathway and the TGF-β signaling pathway (*P* < 0.05, Fig. 4C). These results suggest that lncRNAs may act on protein-coding genes through antisense, *cis* and *trans* modes of action, thereby regulating preadipocyte differentiation in sheep.

### The structure-interaction network of ceRNAs

**Figure 5:**
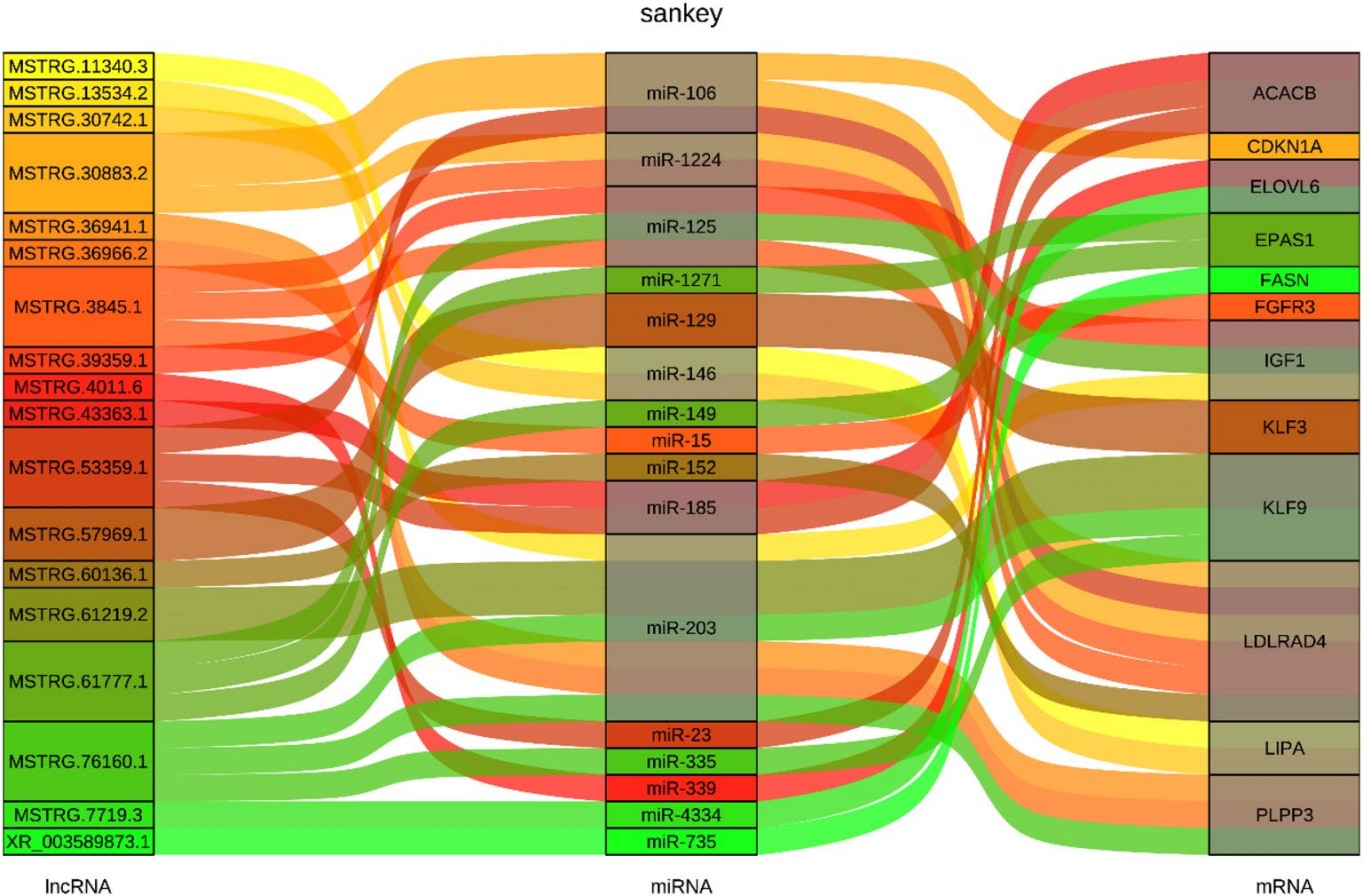
Sankey plot of the lncRNA-miRNA-mRNA network related to ovine preadipocyte differentiation.

This analysis revealed a total of 12,528 lncRNA-miRNA-mRNA binding pairs. That is, 427 differentially expressed lncRNAs would bind 421 miRNAs to increase the expression of 1571 target mRNAs (Supplementary Table 2). The 18 most significant ceRNA relationship pairs from all ceRNAs were chose to construct a network diagram (Fig. 5), and together the above findings suggest that differentially expressed lncRNAs may act as ‘molecular sponges’ for miRNAs during ovine preadipocyte differentiation.

### RT-qPCR verification of the RNA-Seq data

**Figure 6:**
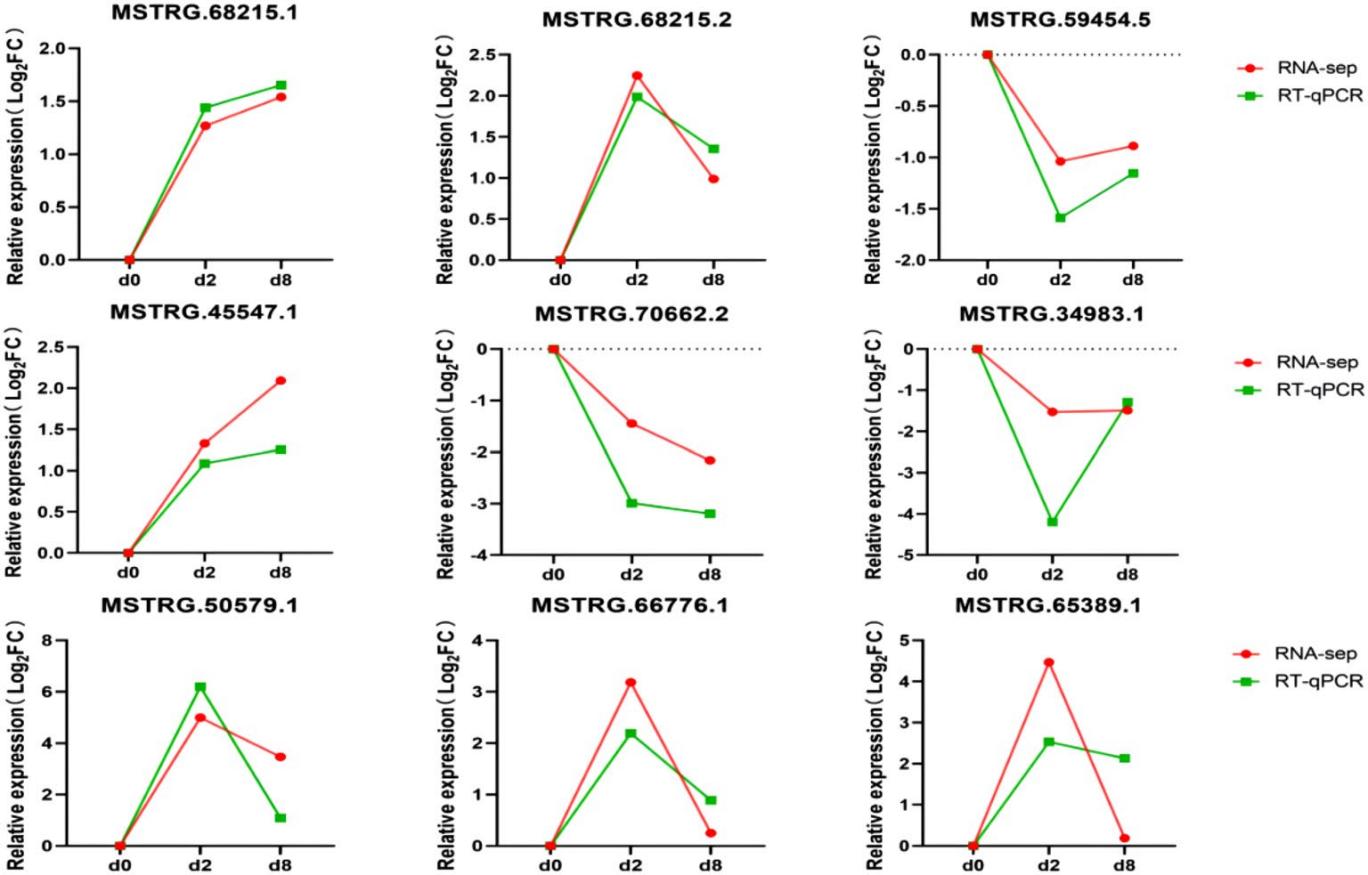
Comparison of RT-qPCR and RNA-Seq results for nine selected lncRNAs.

To verify the RNA-Seq results, nine differentially expressed lncRNAs were selected and analysed using RT-qPCR. The RT-qPCR expression patterns of the selected differentially expressed lncRNAs were found to be consistent with the expression trends from the RNA-Seq results during preadipocyte differentiation (Fig. 6), with this confirming the reliability of the RNA-Seq method used in this study.

## Discussion

In our study, lncRNAs were isolated using an RNA-Seq technology from preadipocytes on day 0, day 2 (early-stage differentiation) and day 8 (late-stage differentiation). The counts of raw reads from the three groups of 12 samples were all over 13.7 Gb, and the high sequencing depth has been illustrated to increase the reliability of sequencing results^22^. The total bases number of filtered high quality data (clean reads) in this study was also over 13.5 Gb.

This high quality and depth of sequencing confirmed the accuracy and reliability of these sequencing data. The mapped ratio of clean reads to the sheep reference genome was 91.65–94.45%, which is consistent with other studies on adipose tissues. For example, 93–95% of adipocyte RNA clean reads in Laiwu pigs can be aligned with the reference genome^23^ and more than 95.9% of the clean reads from the adipose tissue of Hanwoo cattle can be mapped to reference genome^24^.

A total of 2517 novel lncRNAs and 3943 known lncRNAs were identified in the study, which is consistent with what has been reported for similar analyses of other sheep tissues. For example, Ren et al.^25^ identified 6924 lncRNA transcripts associated with skeletal muscle development in Hu sheep.

In the present study, the greatest number of lncRNAs were identified from the intergenic region, with 2445 and 1388 known and novel lncRNAs revealed respectively. This is similar to what has been reported in a previous study^26^, which found 2120 novel lncRNAs in chicken intramuscular preadipocytes.

In this study, it was found that most lncRNAs showed a medium to low abundance expression pattern. Accordingly, it might be speculated that these lncRNAs influence the preadipocyte differentiation process by interacting with related genes during the differentiation of the cells. Of the co-expressed lncRNAs, the most abundant was MSTRG.65945.1. The lncRNA is transcribed from ovine chromosome 20, and the target gene prediction analysis, suggested that MSTRG.65945.1 would *trans*-regulate the fatty acid synthase gene (*FASN*; Chromosome 11), the perilipin 2 gene (*PLIN2*; Chromosome 2) and the Krüppel-like factor 4 gene (*KLF4*; Chromosome 2).

It has been reported that fatty acid synthase can increase lipid accumulation in adipocytes by controlling triglyceride synthesis and degradation^27^. Over-expression of *PLIN2* increased lipid accumulation, whereas knockdown of *PLIN2* prevented lipid accumulation in goat mammary epithelial cells^28^. Xu et al.^29^ found that overexpression of *KLF4* suppressed expression of *C/EBPβ* and *PPARG* in goat muscle preadipocytes, thereby inhibiting preadipocyte differentiation, and another study revealed that overexpression of PPARG induces the accumulation of triglycerides and the up-regulation of *PLIN2*^30^, hence linking the activity of *PLIN2* and *KLF4*. Together, this suggests that MSTRG.65945.1 may play a role in ovine preadipocyte differentiation through regulation of the activity of *FASN*, *PLIN2* and *KLF4*, but the specific functions of most of the lncRNAs identified in this study remain unclear, and their roles in ovine preadipocyte differentiation need to be further explored.

A total of 405, 272 and 359 differentially expressed lncRNAs were identified in the comparisons of D0 and D2, D0 and D8 and D2 and D8. The maturation of preadipocytes is reported to involve several biological processes, including proliferation, apoptosis, and differentiation^4^, hence the differentially expressed lncRNAs may contribute to the different processes occurring in the ovine preadipocytes at the different stages of differentiation. This too will require further detailed investigation. In this respect, numerous studies have shown that the transcriptional regulatory functions of lncRNAs are achieved by acting on protein-coding genes. In this study, the functional analysis revealed that the target genes of the differentially expressed lncRNAs were annotated to biological processes related to enzyme activity and immunological aspects by antisense action. Through *cis*-action, the target genes were annotated to biological processes related to transporter protein activity and catabolic processes, and through *trans*-action, the target genes were annotated to important biological processes related to ovine preadipocyte differentiation, such as receptor binding, adipocyte differentiation, cell proliferation apoptosis and cell differentiation processes.

Specific examples include MSTRG.41060.2, MSTRG.66777.1, MSTRG.13516.1 and MSTRG.39279 targeting the genes *ENPP2*, *NEDD9*, *FGF2* and *SMAD7*, respectively, which are enriched in the regulation of TGF-β receptor signaling pathway, cell differentiation, and epithelial cell differentiation, respectively. Mu et al.^31^ found that *ENPP2* was significantly enriched into GO enrichment, and the KEGG pathway related to lipid metabolism, and it was therefore inferred that it was more likely to regulate milk fat metabolism. A study has found that the lack of *ENPP2* has a significant protective effect on hepatic steatosis, suggesting a possible role of *ENPP2* in the metabolism of milk lipids^32^. The gene *NEDD9* can maintain the expression of hexokinase to promote glycolysis, which provides the necessary energy for the maturation process of preadipocytes^33^, and another study has revealed that *FGF2* stimulates subcutaneous adipose stem cells to promote lipogenic differentiation by activating thePI3K/Akt signaling pathway^34^. The miR-181a-5p promotes 3T3-L1 preadipocyte differentiation by directly targeting *SMAD7* and *TCF7L2* to regulate TGFβ/SMAD and Wnt signaling pathways^35^. Taken together, this strongly suggests the lncRNAs identified here, may be involved in ovine preadipocyte differentiation through *trans*-regulation of target genes.

In the KEGG enrichment analysis, it was found that differentially expressed lncRNAs exerted antisense or *cis-*regulatory effects on signaling pathways, including fatty acid degradation, taurine and hypotaurine metabolism, and glycerolipid metabolism. Fatty acid degradation and glycerolipid metabolism are both major functions of adipocytes, and taurine chloramine may inhibit the differentiation of preadipocytes to adipocytes^36^. Together, these results suggest that differentially expressed lncRNAs may also play a role in preadipocyte differentiation through antisense and *cis*-activation.

The *trans* target genes of differentially expressed lncRNAs were enriched in the MAPK signaling pathway, the PI3K-Akt signaling pathway and the TGF-β signaling pathway. It has been reported that differentiation can be promoted in porcine adipocytes via the MAPK signaling pathway, which has been revealed to inhibit early adipogenesis in 3T3-L1 adipocytes^37,38^. The extract of Alchemilla monticola and Ziziphus jujuba Mill can inhibit adipocyte differentiation by suppressing PI3K-Akt signaling pathway^39,40^. Li et al.^41^ revealed that the TGF-β/SMAD signaling pathway has a negative regulatory effect on lipogenic differentiation. These results suggest that lncRNAs play an important regulatory role in ovine preadipocyte differentiation through *trans*-regulatory effects.

The 18 most significant ceRNA relationship pairs were selected to construct a network diagram (Fig. 5). Among these possible ceRNA regulatory pathways, it was found that some lncRNAs may regulate the expression of the target genes related to adipogenesis through miRNAs. For example, lncRNA MSTRG 61777.1 would regulate *EPAS1* via miR-149, lncRNA XR_003589873.1 would regulate *FASN* via miR-735, and lncRNA MSTRG.53359.1 would affect *LDLRAD4* expression level via miR-106. Previous studies have shown that these target genes play crucial roles in preadipocyte differentiation and lipogenesis^27,42^.

## Materials and methods

### Ethics declarations

All experiments on these sheep were conducted according to animal protection and use guidelines established by the Animal Care Committee at Gansu Agricultural University (Approval number GSAU-Eth-AST-2021-027).

### Sample collection, cell culture and induced differentiation of ovine preadipocytes

Ovine preadipocytes were derived from subcutaneous fat from the groin of three 1.5-year-old Tibetan rams (A sheep breed form the Qinghai-Tibetan Plateau at altitudes above 3000 m), according to our previous reports^43–45^. Specifically, after removing visible blood vessels and connective tissue, adipose tissue samples from the three Tibetan rams were pooled and minced under sterile conditions into small pieces of approximately 1.0 mm^3^. Subsequently, approximately 1 g of minced adipose tissue was added to 20 mL of tissue digestion solution, consisting of 0.75 U/mL collagenase IV solution (Invitrogen, CA, USA), and 1.0 U/mL neutral protease II solution (Invitrogen, CA, USA), and incubated at 37 °C for 1 h. The adipose tissue was digested into a cell suspension by stirring at 37 °C for 1 h. Ovine preadipocytes were isolated from the mixtures by centrifugation at 1500×*g* for 5 min, and then they were resuspended in growth medium. The ovine preadipocytes were then cultured in a growth medium containing 10% fetal bovine serum (Gibco, NY, USA) and DMEM/F12 at 37 °C and in an atmosphere of 5% CO_2_.

Differentiation inducers were used to induce differentiation of the ovine preadipocytes, and they included 1 μg/mL insulin, 0.1 μg/mL dexamethasone, and 27.8 μg/mL 3-isobutyl-1-methylxanthine. After 2 days of inducement, the preadipocytes were further differentiated in a maintenance medium (growth medium and 1 μg/mL insulin). After another 2 days, the maintenance medium was replaced with the growth medium without the insulin until differentiation was completed. The differentiation process described above lasted for 8 days in total. According to the differentiation models described in previous studies, we accordingly collected cells from days 0 (D0), 2 (D2), and 8 (D8) of the differentiation of ovine preadipocytes, respectively. We then harvested and used these cells to extract total RNA for RNA-Seq analysis and four biological duplicates were established at each period.

### RNA extraction, library construction and sequencing

Total RNA was extracted using the Trizol reagent kit (Invitrogen, Carlsbad, CA, USA), and RNA quality was assessed using an Agilent 2100 Bioanalyzer (Agilent, CA, USA). Subsequently, rRNA was eliminated, but mRNA and non-coding RNA (ncRNA) were kept. With the use of fragmentation buffer and random primers, the enriched mRNA and ncRNA were broken up into small pieces and then reverse transcribed into cDNA. Using DNA polymerase I, RNase H, dNTP (dUTP instead of dTTP), and buffer, a second-strand cDNA was created. The cDNA fragments were then purified and end-repaired. They were poly (A) tailed and ligated to Illumina sequencing adapters using the QiaQuick PCR kit (Invitrogen, CA, USA). The UNG (uracil-n-glycosylase) enzyme was used to digest the second-strand cDNA. The digested products were analyzed using agarose gel electrophoresis, and then sequenced using Illumina HiSeqTM4000 sequencing by Gene Denovo Biotechnology (Guangzhou, China).

### The identification of lncRNAs

**Figure 7:**
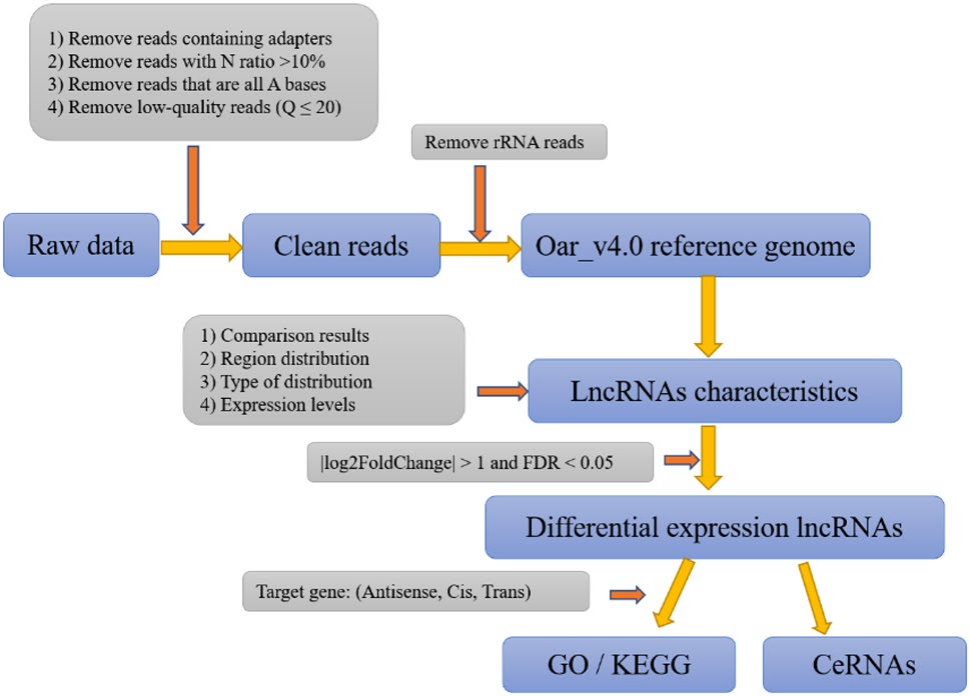
A diagram of the data analysis workflow.

The original data was filtered to assure data quality and accuracy. First, the low-quality data were filtered from the raw data using fastp^46^ to leave clean reads. Next, the clean data's Q20, Q30, GC-content, and sequence duplication levels were determined. Using Bowtie2 v2.2.8, the clean reads were further mapped to the rRNA database to eliminate mapped rRNA reads. Finally, the clean reads were mapped to the ovine Oar_v4.0 reference genome using Hisat2 v2.1.0 (Fig. 7).

Transcripts that were revealed by the sequencing result but were un-annotated in the ovine reference genome, were defined as novel transcripts. Transcripts were eliminated if they were of a length of less than 200 nucleotides in length, or the only represented one exon of a gene. The remaining transcripts with coding capacity were identified using CNCI v2.0^47^ and CPC v 0.9-r2^48^ software, and the outputs from the two programs were combined. These transcripts were regarded as new lncRNAs since their coding potential score was less than 0. Meanwhile, the types, transcript lengths, exon counts and coding potential scores of the new transcripts were also analyzed using Perl scripts (https://www.perl.org/).

### Analysis of the differential expression of lncRNAs

The expression level of lncRNAs was normalized by calculating the fragments per kilobase of transcript per million reads mapped (FPKM) using Stringtie v1.3.4. The lncRNAs with FPKM > 0.1 were considered to be meaningfully expressed. Next, DESeq v2.0 was used to analyze the differential expression of lncRNAs at the three stages of differentiation. These lncRNAs with |log2FoldChange|> 1 and *FDR* < 0.05 were concluded to be the significant differentially expressed lncRNAs. Heat maps of differentially expressed lncRNAs were analyzed using the z-score method according to the FPKM values^21^, and the results were displayed using omicshare platform (https://www.omicshare.com/tools/Home/Soft/getsoft).

### GO and KEGG analyses of the target genes of lncRNAs

To explore the role of the lncRNAs in the differentiation of ovine preadipocytes, the target genes of the lncRNAs were predicted based on the antisense, *cis* and *trans* principle. We also predicted the complementary binding between antisense lncRNAs and mRNAs using RNAplex^49^ and ViennaRNA^50^ package. *Cis* target genes were searched within the range of 10 kb from the lncRNAs. *Trans*-action meant that there were complementary sequences shared between the mRNAs and lncRNAs. Moreover, the sequences of mRNAs that overlapped with lncRNAs were predicted by LncTar software^51^. These three interaction mechanisms were considered preferential for the prediction of the lncRNA-targeted genes. Gene functional enrichment analysis included GO (Gene Ontology; http://www.geneontology.org/), and KEGG (Kyoto Encyclopedia of Genes and Genomes; http://www.genome.jp/kegg/), which were utilized for screening the liposynthesis-related genes associated with the differentially expressed lncRNAs.

### A ceRNA network of lncRNA-miRNA-mRNA

Based on our previously published ovine preadipocyte mRNA data (SRR19917845-SRR19917856) and miRNA data from the same samples as those used in the study^43,44^, a lncRNA associated ceRNA network was constructed according to the ceRNA construction method established by Shen et al.^52^. (1) Spearman rank correlation coefficient (SCC) was used to evaluate the expression association between mRNA-miRNA or lncRNA-miRNA. The pairs with SCC > 0.7 were selected as negatively co-expressed lncRNA-miRNA pairs or mRNA-miRNA pairs. Both mRNAs and lncRNAs were considered to be the target genes of miRNAs. (2) The Pearson correlation coefficient (PCC) was used to assess the expression relationship between lncRNAs and mRNAs. The pairs with PCC > 0.9 were selected as co-expressed lncRNA-mRNA pairs. (3) A hypergeometric cumulative distribution function test was performed to determine whether the shared miRNA sponges between the two genes were noteworthy. Only gene pairs with a *P*-value < 0.05 were selected. Finally, Cytoscape v3.5.1 was used to display a lncRNA-miRNA-mRNA network.

### Validation of differentially expressed lncRNAs and statistical analysis

Nine differentially expressed lncRNAs were randomly selected from three periods of ovine preadipocytes differentiation and subjected reverse transcription-quantitative PCR (RT-qPCR) to establish the RNA expression levels. Specifically, the HiScript II Q RT SuperMix for qPCR (+ gDNA wiper) kit (Vazyme Biotech, Xuanwu Qu, China) was used to synthesize first-strand cDNA with *GAPDH*^26,53^ being employed as an internal reference gene. The original RNA samples used for the RNA-Seq were also used as the templates for the reverse transcription reactions in the RT-qPCRs. The results were calculated using the 2^-ΔΔCt^ method to verify the reliability of RNA-Seq sequencing data.

The primer information of the selected lncRNAs and the internal reference gene is shown in Table 2.

**Table 2:** Information of primer sequence.

Name	Forward primer sequence (5′ → 3′)	Reverse primer sequence (5′ → 3′)
*MSTRG.68215.1*	AGTGCTCCCCTTTGGACTTT	ACTTTCCCGGCTTACCAGAT
*MSTRG.68215.2*	CCTGTGTGAGGTGGGAGAAT	GCCCAGAGAAGGTGAGTGAG
*MSTRG.59454.5*	AGTAGCCAGTCCCCTCGTCT	CGGGTCCTTGTTAGGTTTGA
*MSTRG.70662.2*	CTTTTCCCCACTGCGTTTTA	CACATGGAGTAGGGCACCTT
*MSTRG.34983.1*	TGTGGAGCTGTTCTGCAATC	GCACAAGGAATCAGCAGACA
*MSTRG.50579.1*	TCTGGACCCTGTCAACATCA	CCCACACCACACAGCATAAG
*MSTRG.66776.1*	CTGCCAAGCCCTAAAGTGAG	CTTGACTCCGACTCCCTCAG
*MSTRG.65389.1*	ACTTGACGGACCTTTGTTGG	TCTTGGGCTCCAGAATGACT
*MSTRG.45547.1*	CTGCACTCACCATTTGCACT	CGCAGATAACACCACGCTTA
*GAPDH*	CACAGTCAAGGCAGAGAACG	CAGCCTTCTCCATGGTAGTG

## Conclusions

This study described the lncRNA expression profiles of ovine preadipocytes at three stages of differentiation. A total of 2517 novel lncRNAs and 3943 known lncRNAs were found to be expressed, and a total of 405, 272 and 359 differentially expressed lncRNAs were found in the D0-vs-D2, D0-vs-D8, and D2-vs-D8 preadipocytes respectively. The differentially expressed lncRNAs were enriched in the signaling pathways related to ovine preadipocytes differentiation, such as MAPK, PI3K-Akt, and TGF-β. This study provides an improved understanding of the roles of lncRNAs in ovine preadipocytes differentiation.

### Supplementary Information

Supplementary Information 1.

Supplementary Information 2.

Supplementary Information 3.

## Data Availability

The data in the study has been saved in GenBank with accession numbers SRR19917845-SRR19917856.
