# Supplementary material for: Screening and identification of lncRNAs in preadipocyte differentiation in sheep
Source: Sci Rep. 2024 Mar 4;14:5260. doi: 10.1038/s41598-024-56091-5 (PMC10912770; doi:10.1038/s41598-024-56091-5)
Supplement: Supplementary file 3 — Supplementary Information 3. [file 41598_2024_56091_MOESM3_ESM.docx]

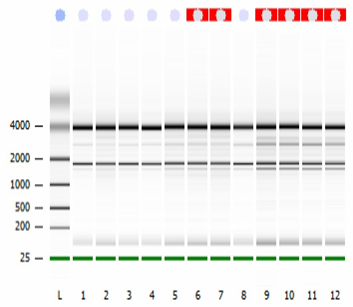


**Fig. S1** The results of total RNA of ovine adipocytes by RNA Agilent 2100 analysis.

(Note: L: DNA Marker 5000; 1-9 represents the sample of D0-1, D0-2, D0-3, D0-4, D2-1, D2-2, D2-3, D2-4, D8-1, D8-2, D8-3 and D8-4).

Table S1 Agilent 2100 analysis of total RNA isolated from ovine adipocytes.

| Samples | Stock solution concentration（μg/uL） | Total RNA（μg） | RIN |
| --- | --- | --- | --- |
| D0-1 | 256 | 10.87 | 9.8 |
| D0-2 | 301 | 11.14 | 9.5 |
| D0-3 | 278 | 12.23 | 9.7 |
| D0-4 | 305 | 12.20 | 9.8 |
| D2-1 | 287 | 10.63 | 9.1 |
| D2-2 | 319 | 11.08 | 9.4 |
| D2-3 | 321 | 12.20 | 9.5 |
| D2-4 | 348 | 15.31 | 9.5 |
| D8-1 | 417 | 15.43 | 9.4 |
| D8-2 | 402 | 15.68 | 9.6 |
| D8-3 | 438 | 19.27 | 9.1 |
| D8-4 | 482 | 20.73 | 9.2 |
